# Medium Term Outcomes of Revision Laparoscopic Sleeve Gastrectomy after Gastric Banding: A Propensity Score Matched Study

**DOI:** 10.1007/s11695-023-06629-9

**Published:** 2023-05-22

**Authors:** Brenda W. Huang, Sarfraz S. Shahul, Marcus K.H. Ong, Oliver M. Fisher, Daniel L. Chan, Michael L. Talbot

**Affiliations:** 1 Faculty of Medicine, University of New South Wales, 18 High St, Kensington, NSW 2052 Australia; 2 Upper Gastrointestinal Surgery Unit, Department of Surgery, St George Hospital, Gray St, Kogarah, NSW 2217 Australia; 3 School of Medicine, Western Sydney University, Narellan Rd & Gilchrist Dr, Campbelltown, NSW 2560 Australia

**Keywords:** revisional sleeve gastrectomy, primary sleeve gastrectomy, weight loss outcome, complications

## Abstract

**Purpose:**

Revision bariatric surgery may be undertaken after weight loss failure and/or complications following primary bariatric surgery. This study aims to compare the efficacy and safety of revision laparoscopic sleeve gastrectomy (RLSG) after gastric banding (GB) to those of primary laparoscopic sleeve gastrectomy (PLSG).

**Materials and Methods:**

A retrospective, propensity-score matched study was conducted to compare between PLSG (control) patients and RLSG after GB (treatment) patients. Patients were matched using 2:1 nearest neighbor propensity score matching without replacement. Patients were compared on weight loss outcomes and postoperative complications for up to five years.

**Results:**

144 PLSG patients were compared against 72 RLSG patients. At 36 months, PLSG patients had significantly higher mean %TWL than RLSG patients (27.4 ± 8.6 [9.3–48.9]% vs. 17.9 ± 10.2 [1.7–36.3]%, *p* < 0.01). At 60 months, both groups had similar mean %TWL (16.6 ± 8.1 [4.6–31.3]% vs. 16.2 ± 6.0 [8.8–22.4)]%, *p* > 0.05). Early functional complication rates were slightly higher with PLSG (13.9% vs. 9.7%), but late functional complication rates were comparatively higher with RLSG (50.0% vs. 37.5%). The differences were not statistically significant (*p* > 0.05). Both early (0.7% vs 4.2%) and late (3.5% vs 8.3%) surgical complication rates were lower in PLSG patients compared to RLSG patients but did not reach statistical significance (*p* > 0.05).

**Conclusion:**

RLSG after GB has poorer weight loss outcomes than PLSG in the short-term. Although RLSG may carry higher risks of functional complications, the safety of RLSG and PLSG are overall comparable.

**Graphical abstract:**

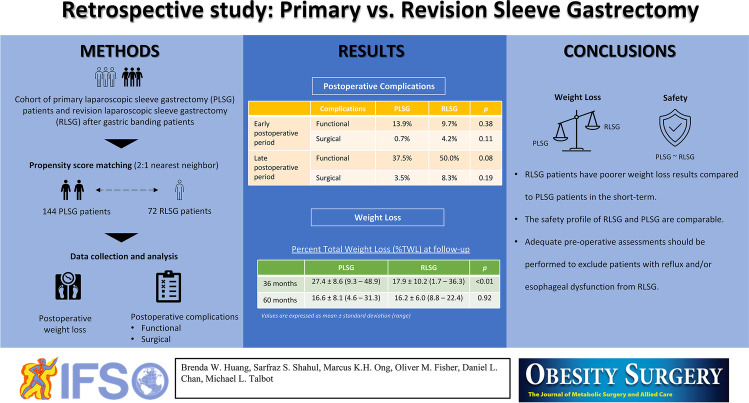

## Introduction

Gastric banding (GB) was once a popular procedure known for its simplicity and adjustability. GB is now less frequently performed due to poor long-term weight maintenance and associated band complications, which have necessitated revision surgery in up to 60.0% of patients [1–4]. Whilst revision bariatric surgery may yield further weight loss (WL), it carries inherent risks as intra-abdominal adhesions and anatomical changes after primary GB will increase technical complexity [5, 6].

Options following failed GB include band replacement or conversion to other bariatric procedures. Whilst band replacement may resolve some complications, procedures which offer a broader spectrum of WL mechanisms, such as revision gastric bypass and revision laparoscopic sleeve gastrectomy (RLSG) are preferred [7–9]. There have been recent trends towards RLSG after GB failure, given its comparable WL results [10–16]. Compared to the complication risks of revision gastric bypass, the complication risks of RLSG are similar in some studies [11, 16, 17] but lower in other studies [13, 15, 18, 19].

Although the results of RLSG after GB are promising, it is uncertain how they compare to those of primary laparoscopic sleeve gastrectomy (PLSG). Few comparative studies have assessed RLSG’s outcomes relative to PLSG’s, but the results were disparate and limited by poor follow-up and confounding biases. This study aimed to compare RLSG after GB to PLSG in terms of safety and WL efficacy in the short and medium term, using propensity score matching (PSM) to reduce bias.

## Material and Methods

### Study Design

A single-center, retrospective, propensity-score matched study was performed on patients who underwent RLSG after GB (treatment) and patients who underwent PLSG (control) from 2005 to 2019 at an Australian Metabolic and Bariatric Surgery Centre of Excellence. These patients were identified through electronic medical records via billing code searches. Patients who underwent RLSG for insufficient weight loss (IWL) or weight regain (WR) after GB with or without band-related complications were included. IWL is defined as less than 50% excess weight loss (%EWL) 18 months postoperatively, and WR is progressive weight gain after an initial successful weight loss (>50%EWL) [20]. Patients who underwent RLSG for complications only were excluded. Patients were offered high resolution impedance manometry before RLSG; those with major peristaltic abnormalities, ineffective motility or pseudo-achalasia were offered Roux-en-Y gastric bypass as a preferred alternative. Patients with incomplete follow-up (<6 months post PLSG or RLSG) and history of other bariatric procedures before PLSG or RLSG, or major abdominal surgery were excluded.

Patient demographics, preoperative and postoperative anthropometric data, and postoperative complications were recorded. Postoperative complications were categorized based on time frame (early and late) and type (functional and surgical). Functional complications included dysphagia and new-onset reflux. Postoperative reflux was documented based on patient symptomatology, using the Visick scoring system [21]. Surgical complications included leaks and surgical site infections. Postoperative weight and body mass index (BMI) were collected to calculate change in BMI (ΔBMI), %EWL and percent total weight loss (%TWL), following the American Society for Metabolic and Bariatric Surgery guidelines [22].

Two PLSG patients were matched to one RLSG patient using nearest neighbor PSM without replacement (caliper = 0.1). Patients were matched for age, sex, smoking history, preoperative BMI, and associated medical problems (diabetes and gastroesophageal reflux disease [GERD]). PSM was performed on R software, version 4.0 (R Foundation for Statistical Computing, Vienna, Austria) with “MatchIt” package.

Following the Shapiro-Wilk test for normality, parametric data were presented as mean ± standard deviation (range) and nonparametric data as median (range). Parametric and nonparametric data were analyzed using independent samples *t*-test and Mann-Whitney U test, respectively. Categorical data were presented as counts with corresponding percentages (%) and analyzed using Pearson’s Chi-square test and Fisher’s exact test, where appropriate. All tests were 2-tailed and statistically significant if *p* < 0.05. Statistical analyses were performed using IBM SPSS Statistics for Windows, version 26.0 (IBM Corp., Armonk, N.Y., USA).

This project was approved by the institutional human research advisory panel (approval number HC190695).

### Procedure selection and pre-operative assessment

Patients presenting for the management of obesity at the time of this study were offered laparoscopic adjustable GB, laparoscopic sleeve gastrectomy and or gastric bypass depending on patient preference and the presence of any contraindications to various procedures. The consent process involved shared decision making, whereby the preferences of the patient were taken into account. Severe reflux, diabetes, and BMI >50, for example, were reviewed as relative indications for gastric bypass ahead of other procedures. However, absolute contraindications to particular therapies were uncommon. Oesophageal dilation, Barrett’s oesophagus and large hiatus hernia were absolute contraindications to laparoscopic sleeve gastrectomy and laparoscopic adjustable GB, whereas large ventral hernia and complex intra-abdominal adhesions were indications against gastric bypass unless the patient was prepared to undergo laparotomy.

PLSG patients did not undergo pre-operative endoscopy unless they had proton-pump inhibitor resistant reflux symptoms or dysphagia. The large majority of patients undergoing RLSG underwent 2-stage surgery (band removal followed by sleeve gastrectomy in two separate operations), which allowed clinical evaluation for reflux symptoms or post-GB oesophageal complications by high resolution impedance manometry after band removal or a contrast swallow if manometry was refused or not tolerated. Manometry and/or contrast swallows were also undertaken in the small group of patients undergoing 1-stage RLSG (band removal and sleeve gastrectomy in a single operation). Gastroscopy was also selectively undertaken in this cohort.

### Surgical Technique

Under general anesthesia, the patient was positioned supine in reverse Trendelenburg position. Four laparoscopic ports and a Nathanson liver retractor (Cook Medical) were utilized. The esophageal hiatus was routinely inspected; if a hiatal hernia were detected, it was repaired anteriorly or posteriorly with a non-absorbable suture. An adhesiolysis and excision of GB pseudocapsule were performed to restore normal anatomy before gastric transection. The greater curvature of the stomach was mobilized from the pylorus to the hiatus, ensuring complete mobilization of the fundus. The stomach was then transected, typically using 6 x 60 mm laparoscopic staple firings over a 36 French bougie. Gastric division started 2-4 cm from the pylorus with the stapler kept deliberately wide of the bougie until beyond the angularis to prevent creating a functional stenosis. After completing the staple line and obtaining hemostasis, the gastric tube was sutured lightly to omental tissue for stabilization. The resected portion of stomach was removed via the right lateral port site before wound closure.

## Results

**Fig. 1:**
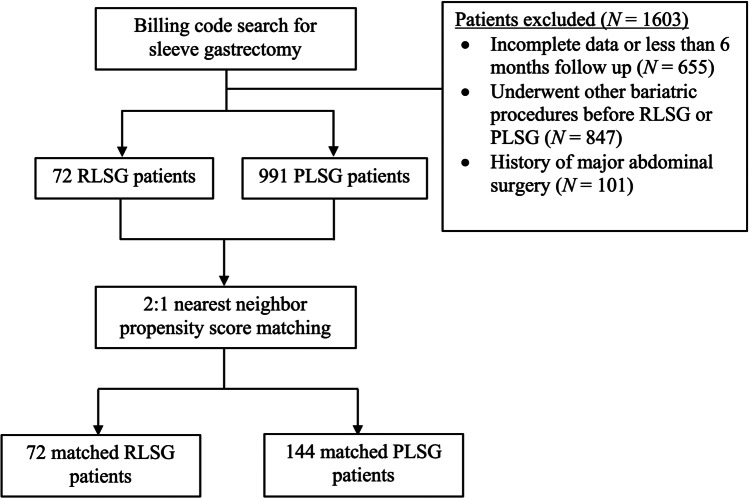
Flowchart of patient selection for primary laparoscopic sleeve gastrectomy and revision laparoscopic sleeve gastrectomy groups. *PLSG* primary laparoscopic sleeve gastrectomy; *RLSG* revision laparoscopic sleeve gastrectomy; *N* number of patients

**Fig. 2:**
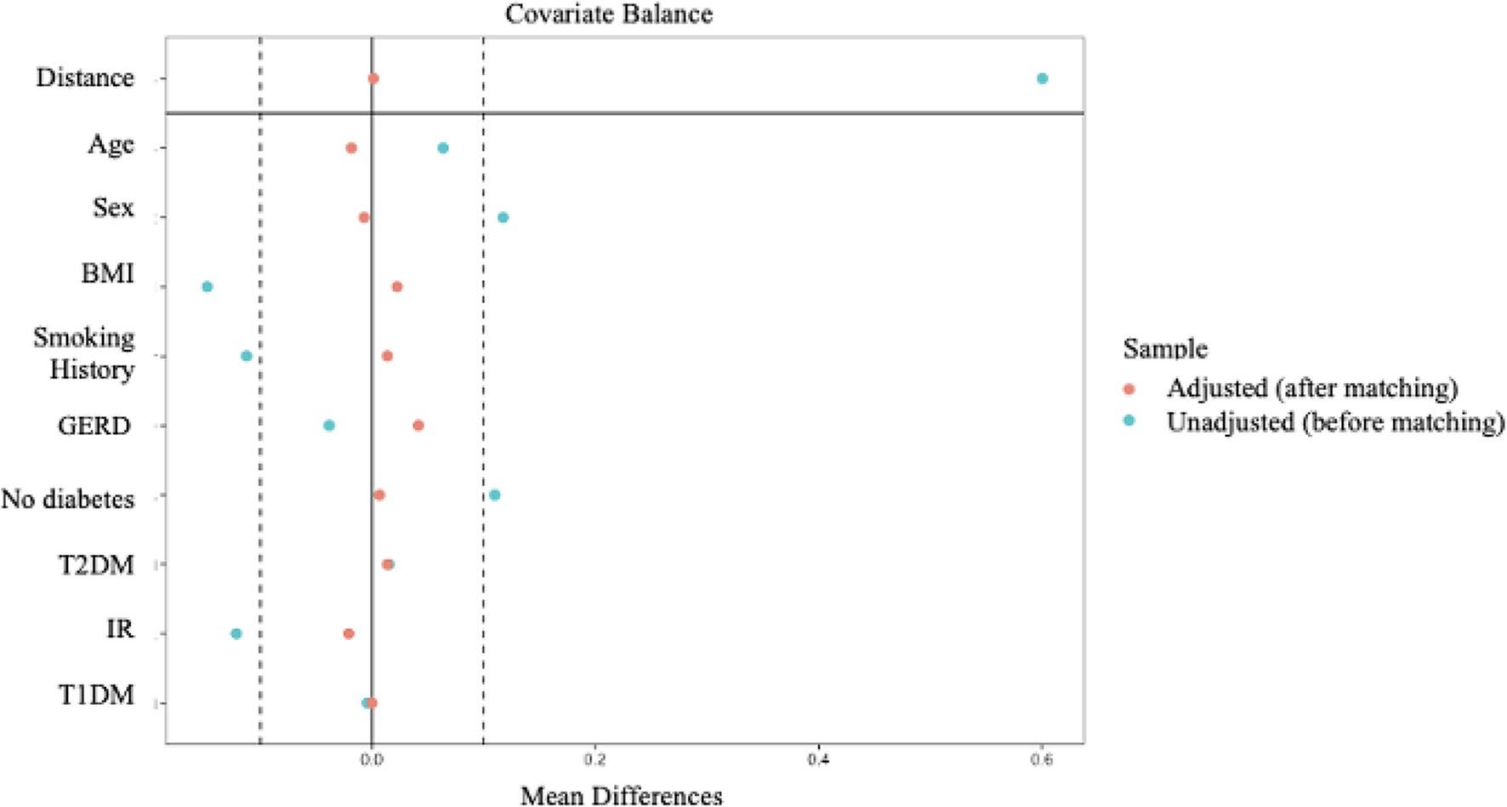
Covariate balance before and after matching for primary and revision laparoscopic sleeve gastrectomy groups. *BMI* body mass index; *GERD* gastroesophageal reflux disease; *T2DM* Type 2 diabetes mellitus; *IR* insulin resistance; *T1DM* Type 1 diabetes

991 PLSG patients and 72 RLSG patients met the inclusion criteria (Fig. 1). A control group of 144 PLSG patients were selected using nearest neighbor PSM without replacement (ratio = 2:1; caliper = 0.1) (see Appendix, Table 6). The covariate balance before and after PSM is demonstrated in Fig. 2; the standardized mean differences for all covariates were below the threshold of 0.1 after PSM. Both groups were similar in baseline characteristics (Table 1) except for a higher prevalence of dyslipidemia amongst PLSG patients.

**Table 1:** Baseline characteristics of patients in the primary laparoscopic sleeve gastrectomy and revision laparoscopic sleeve gastrectomy groups

	Overall	Patient groups		*p*
		PLSG	RLSG	
*N*	216	144	72	
Age, years*	45.5 ± 11.8 (18.4 – 75.4)	45.5 ± 11.9 (18.4 – 74.5)	45.3 ± 11.7 (23.1 – 75.4)	0.90
Female gender, *n* (%)	187 (86.6%)	125 (86.8%)	62 (86.1%)	0.89
Preoperative BMI, kg/m^2^*	42.6 ± 7.0 (30.1 – 69.2)	42.5 ± 6.5 (30.2 – 68.2)	42.7 ± 7.9 (30.1 – 69.2)	0.86
Smoking history** (*n* = 183)	64 (29.6%)	42 (29.2%)	22 (30.6%)	0.35
Associated Medical Problems				
GERD, *n* (%)	69 (31.9%)	44 (30.6%)	25 (34.7%)	0.54
Type 2 diabetes mellitus, *n* (%)	34 (15.7%)	22 (15.3%)	12 (16.7%)	0.31
Hypertension, *n* (%)	77 (35.6)	54 (37.5%)	23 (31.9%)	0.42
Dyslipidemia, *n* (%)	69 (31.9%)	53 (36.8%)	16 (22.2)	0.03
Obstructive sleep apnea, *n* (%)	31 (14.5%)	23 (16.0%)	8 (11.4%)	0.38

*N* number of patients; *BMI* body mass index; *GERD* gastroesophageal reflux disease; *PLSG* primary laparoscopic sleeve gastrectomy; *RLSG* revision laparoscopic sleeve gastrectomy

*Values are expressed as mean ± standard deviation (range)

**Data on smoking history were only available for 183 patients

Within the RLSG group, 13 (18.1%) patients underwent revision for IWL or WR only while 59 (81.9%) underwent revision for IWL or WR and band-related complications (Table 2). Prerevision WL data were available for 50 RLSG patients, who had a mean %TWL of 23.4 ± 10.5 (2.1–57.9) % at nadir BMI post-GB. %TWL decreased to 5.9 ± 9.9 (-24.4–29.0) % at revision. All but one RLSGs were conducted in 2 stages; the median time interval between band removal and RLSG was 3.8 (2.1–57.4) months.

**Table 2:** Characteristics of patients in the revision laparoscopic sleeve gastrectomy group

Variables	Statistics
Anthropometric data (*N* = 50)	
Initial BMI before GB, kg/m^2^*	44.6 ± 6.6 (30.9 – 61.1)
Nadir BMI after GB, kg/m^2^*	33.8 ± 6.1 (22.4 – 51.6)
%TWL at nadir BMI after GB*	23.4 ± 10.5 (2.1 – 57.9)
%TWL at time of revision*	5.9 ± 9.9 (-24.4 – 29.0)
Band Complications (*N* = 59)	
Dysphagia/ food intolerance, *N* (%)	27 (45.8%)
Pouch dilatation, *N* (%)	16 (27.1%)
GERD, *N* (%)	16 (27.1%)
Esophageal dilatation, *N* (%)	11 (18.6%)
Band slippage, *N* (%)	10 (16.9%)
Band erosion, *N* (%)	4 (6.8%)

*N* number of patients*; BMI* body mass index; *%TWL* percent total weight loss; *GERD* gastroesophageal reflux disease

*Values are expressed as mean ± standard deviation (range)

All PLSGs and RLSGs were performed laparoscopically. Hiatal hernia repair was performed in 71 (49.3%) PLSG cases and 37 (51.4%) RLSG cases. The follow-up rates were 79.2%, 79.2%, 62.0%, 37.5%, 32.9%, 22.2%, 13.9% and 7.9% at 3-, 6-, 12-, 24-, 36-, 48-, and 60-months, respectively.

### Postoperative Complications

Postoperative complications in this study are listed in Table 3. The overall rates of surgical complications between PLSG patients and RLSG patients were not significantly different in the early (<30 days) postoperative period (0.7% vs 4.2%, *p* = 0.11) and in the late (>30 days) postoperative period (3.5% vs. 8.3%, *p* = 0.19). There were two staple line leaks in the RLSG group, which presented as intra-abdominal abscesses. One patient was managed with computed tomography-guided drainage. The other patient previously had band erosion; the leak was initially managed using endoscopic clips but recurred in the late postoperative period and required computed tomography-guided drainage.

**Table 3:** Complications after primary laparoscopic sleeve gastrectomy and revision laparoscopic sleeve gastrectomy

	Overall	Patient groups		*p*
		PLSG	RLSG	
*N*	216	144	72	
Early functional complications, *N* (%)	**27 (12.5%)**	**20 (13.9%)**	**7 (9.7%)**	**0.38**
New-onset reflux	10 (4.6%)	10 (6.9%)	0 (0%)	0.03
Dysphagia	19 (8.8%)	13 (9.0%)	6 (8.3%)	0.87
Required endoscopic dilatation	2 (0.9%)	0 (0%)	2 (3.8%)	0.11
Stricture on endoscopy	1 (0.5%)	0 (0%)	1 (1.4)	0.33
Early surgical complications, *N* (%)	**4 (1.9%)**	**1 (0.7%)**	**3 (4.2%)**	**0.11**
Surgical site infection (conservative management)	2 (0.9%)	1 (0.7%)	1 (1.4%)	1.00
Staple line leak	2 (0.9%)	0 (0%)	2 (2.8%)	0.11
Late functional complications, *N* (%)	**90 (41.7%)**	**54 (37.5%)**	**36 (50.0%)**	**0.08**
New-onset reflux	66 (30.6%)	41 (28.5%)	25 (34.7%)	0.35
Intractable reflux requiring revision gastric bypass	3 (1.4%)	1 (0.7%)	2 (2.8%)	0.26
Dysphagia	40 (18.5%)	25 (17.4%)	15 (20.8%)	0.54
Required endoscopic dilatation	6 (2.8%)	2 (1.4%)	4 (5.6%)	0.10
Stricture at endoscopy	1 (0.5%)	0 (0%)	1 (1.4%)	0.33
Severe nausea and vomiting requiring enteral feeding	1 (0.5%)	0 (0%)	1 (1.4%)	0.33
Late surgical complications, *N* (%)	**11 (5.1%)**	**5 (3.5%)**	**6 (8.3%)**	**0.19**
Hiatal hernia repair	6 (2.8%)	3 (2.1%)	3 (4.2%)	0.40
Incisional hernia repair	4 (1.9%)	2 (1.4%)	2 (2.8%)	0.60
Staple line leak	1 (0.5%)	0 (0%)	1 (1.4%)	0.33

*N* number of patients*; PLSG* primary laparoscopic sleeve gastrectomy; *RLSG* revision laparoscopic sleeve gastrectomy

The rate of functional complications was relatively higher in the PLSG group in the early postoperative period (13.9% vs. 9.7%, *p* = 0.38). However, the rate of functional complications was comparatively higher in the RLSG group in late postoperative period (50.0% vs. 37.5%, *p* = 0.08). Nevertheless, these differences did not reach statistical significance. One RLSG patient developed severe nausea and vomiting, requiring enteral feeding. One RLSG patient had a stricture at the previous band tunnel that required 9 endoscopic balloon dilatations and eventually underwent revision gastric bypass 2.8 years postoperatively due to intractable dysphagia and reflux. One other RLSG patient and 1 PLSG patient also underwent revision gastric bypass after 1.7 and 9.0 years, respectively, due to intractable reflux. There was no mortality in this study.

### Weight Loss

The mean preoperative BMIs were 42.5 ± 6.5 (29.0–68.2) kg/m^2^ in the PLSG group and 42.7 ± 7.9 (30.1–69.2) kg/m^2^ in the RLSG group (*p* = 0.86). Postoperative WL data are listed in Table 4. PLSG patients had greater mean ΔBMI, %EWL and %TWL than RLSG patients at 3, 6, 12, 24 and 36 months (*p* < 0.01). At 48 and 60 months, the differences in ΔBMI, %EWL and %TWL were insignificant (*p* > 0.05). A greater proportion of RLSG patients underwent further revision surgery for WL (12.5% vs. 2.8%, *p* = 0.01).

**Table 4:** Weight loss outcomes after primary laparoscopic sleeve gastrectomy and revision laparoscopic sleeve gastrectomy

Time	Parameters	Overall	Patient Groups		*p*
			PLSG	RLSG	
3 months	*N* (%)	171/216 (79.2%)	115/144 (79.9%)	56/72 (77.8%)	-
	BMI**,** kg/m^2^*	35.5 ± 6.0 (23.9 – 57.9)	34.8 ± 5.5 (23.9 – 54.8)	37.0 ±6.7 (26.8 – 57.9)	0.02
	ΔBMI, kg/m^2^*	7.1 ± 2.7 (-1.3 – 14.9)	7.6 ± 2.0 (3.5 – 13.3)	6.0 ± 3.4 (-1.3 – 14.9)	<0.01
	%EWL*	43.2 ± 18.0 (-26.1 – 122.0)	47.5 ± 16.2 (13.5 – 129.0)	34.3 ± 18.3 (-26.1 – 72.9)	<0.01
	%TWL*	16.5 ± 5.4 (-4.4 – 29.5)	18.0 ± 4.0 (6.8 – 29.5)	13.5 ± 6.6 (-4.4 – 26.9)	<0.01
6 months	*N* (%)	171/216 (79.2%)	118/144 (81.9%)	53/72 (73.6%)	
	BMI**,** kg/m^2^*	33.0 ± 5.7 (22.3 – 52.8)	32.1 ± 5.3 (22.4 – 52.8)	35.0 ± 6.1 (22.9 – 51.5)	<0.01
	ΔBMI, kg/m^2^*	9.8 ± 3.1 (1.6 – 20.0)	10.3 ± 2.9 (4.8 – 20.0)	8.5 ± 3.4 (1.6 – 17.2)	<0.01
	%EWL*	59.3 ± 21.0 (11.5 – 150.9)	63.7 ± 19.5 (31.5 – 150.9)	49.4 ± 19.6 (11.5 – 124.0)	<0.01
	%TWL*	22.7 ± 6.1 (4.1 – 38.3)	24.3 ± 5.3 (11.9 – 38.3)	19.4 ± 6.4 (4.1 – 32.4)	<0.01
12 months	*N* (%)	134/216 (62.0%)	85/144 (59.0%)	49/72 (68.1%)	-
	BMI**,** kg/m^2^*	30.9 ± 5.8 (21.9 – 48.4)	29.9 ± 5.3 (21.9 – 48.4)	32.6 ± 6.1 (22.6 – 48.2)	0.01
	ΔBMI, kg/m^2^*	12.0 ± 4.5 (0.4 – 25.7)	13.0 ± 4.0 (4.2 – 25.7)	10.2 ± 4.8 (0.4 – 21.5)	<0.01
	%EWL*	70.8 ± 23.7 (7.5 – 123.8)	76.8 ± 21.2 (16.4 – 123.8)	60.2 ± 24.3 (7.5 – 121.7)	<0.01
	%TWL*	27.7 ± 8.5 (1.3 – 50.2)	30.2 ± 7.1 (8.3 – 50.2)	23.3 ± 9.1 (1.3 – 46.5)	<0.01
18 months	*N* (%)	81/216 (37.5%)	61/144 (42.4%)	20/72 (27.8%)	-
	BMI**,** kg/m^2^*	31.2 ± 5.8 (22.1 – 47.9)	30.3 ± 5.1 (22.6 – 47.9)	34.1 ± 6.9 (22.1 – 46.9)	0.01
	ΔBMI, kg/m^2^*	12.5 ± 5.2 (-0.25 – 27.6)	13.1 ± 4.7 (2.6 – 27.6)	10.9± 6.3 (-0.3 – 24.7)	0.10
	%EWL*	69.7 ± 24.5 (-1.2 – 117.7)	74.0 ± 22.1 (10.3 – 117.7)	56.6 ± 27.4 (-1.2 – 115.4)	<0.01
	%TWL*	28.3 ± 9.8 (-0.5 – 49.8)	29.9 ± 8.5 (5.2 – 47.7)	23.6 ± 11.8 (-0.5 – 49.8)	0.01
24 months	*N* (%)	71/216 (32.9%)	46/144 (31.9%)	25/72(34.7%)	-
	BMI**,** kg/m^2^*	32.3 ± 6.1 (20.9 – 46.4)	31.0 ± 5.4 (20.9 – 45.5)	34.8 ± 6.6 (23.8 – 46.4)	0.01
	ΔBMI, kg/m^2^*	11.3 ± 5.1 (-2.1 – 29.3)	12.5 ± 4.6 (3.6 – 29.3)	9.1 ± 5.4 (-2.1 – 24.4)	<0.01
	%EWL*	64.6 ± 26.7 (-10.8 – 146.5)	72.0 ± 24.1 (17.2 – 146.5)	51.0 ± 26.5 (-10.8 – 111.2)	0.01
	%TWL*	25.6 ± 9.5 (-4.6 – 45.7)	28.4 ± 7.7 (7.8 – 45.7)	20.3 ± 10.3 (-4.6 – 44.4)	<0.01
36 months	*N* (%)	48/216 (22.2%)	31/144 (21.5%)	17/72(23.6%)	-
	BMI**,** kg/m^2^*	32.1 ± 5.8 (22.9 – 49.0)	30.7 ± 4.4 (23.3 – 39.0)	34.7 ± 7.3 (22.9 – 49.0)	0.05
	ΔBMI, kg/m^2^*	10.4 ± 5.3 (0.8 – 31.3)	11.9 ± 5.1 (3.7 – 31.3)	7.6 ± 4.5 (0.8 – 14.4)	<0.01
	%EWL*	61.9 ± 27.4 (3.9 – 130.9)	69.0 ± 21.2 (24.8 – 111.4)	49.0 ± 32.9 (3.9 – 130.9)	0.01
	%TWL*	24.1 ± 10.2 (1.7 – 48.9)	27.4 ± 8.6 (9.3 – 48.9)	17.9 ± 10.2 (1.7 – 36.3)	<0.01
48 months	*N* (%)	30/216 (13.9%)	18/144 (12.5%)	12/72 (16.7%)	-
	BMI**,** kg/m^2^*	34.2 ± 5.1 (22.5 – 45.0)	34.5 ± 4.9 (25.7 – 45.0)	33.8 ± 5.7 (22.5 – 41.6)	0.75
	ΔBMI, kg/m^2^*	8.3 ± 4.7 (1.0 – 22.2)	9.0 ± 5.4 (1.0 – 22.2)	7.3 ± 3.2 (2.6 – 10.8)	0.04
	%EWL*	49.7 ± 28.4 (8.6 – 137.0)	48.2 ± 25.4 (8.6 – 95.8)	51.8 ± 33.4 (14.2 – 137.0)	0.75
	%TWL*	19.3 ± 9.8 (2.8 – 42.1)	20.3 ± 11.1 (2.8 – 42.1)	17.9 ± 7.9 (6.0 – 29.1)	0.54
60 months	*N* (%)	17/216 (7.9%)	12/144 (8.3%)	5/72 (6.9%)	-
	BMI**,** kg/m^2^*	35.4 ± 3.6 (26.4 – 40.4)	34.9 ± 3.3 (26.4 – 39.4)	36.5 ± 4.2 (29.8 – 40.4)	0.40
	ΔBMI, kg/m^2^*	7.1 ± 3.3 (1.9 – 12.0)	7.1 ± 3.6 (1.9 – 12.0)	7.1 ± 3.0 (3.8 – 11.0)	0.99
	%EWL*	40.5 ± 18.5 (12.2 – 89.3)	41.1 ± 20.1 (12.2 – 89.3)	39.1 ± 16.0 (20.9 – 62.7)	0.85
	%TWL*	16.5 ± 7.3 (4.6 – 31.3)	16.6 ± 8.1 (4.6 – 31.3)	16.2 ± 6.0 (8.8 – 22.4)	0.92

*N* number of patients at a time point out of the total number of patients; *PLSG* primary laparoscopic sleeve gastrectomy; *RLSG* revision laparoscopic sleeve gastrectomy; *BMI* body mass index; *ΔBMI* change in BMI; *%EWL* percent excess weight loss; *%TWL* percent total weight loss

*Values are expressed as mean ± standard deviation (range)

ΔBMI was highest at 18 months follow-up in both groups but decreased subsequently. Similarly, %EWL and %TWL peaked around 12–18 months postoperatively then decreased. This was accompanied by the increase in mean postoperative BMI from 12–60 months follow-up.

## Discussion

PSM is a statistical technique utilized in observational studies to reduce treatment selection bias and better evaluate treatment effects, mimicking the effects of a randomized control trial [23]. In retrospective, non-randomized studies, comparison of treatment effects are difficult as there are confounding factors which have determined treatment assignment. PSM aims to distribute the baseline covariates, which may determine the probability of treatment assignment, between treatment subjects and control subjects [23]. This allows better comparison of outcomes between treatment subjects and control subjects within a cohort. Hence, PSM was used in this study to evaluate the effects of PLSG and RLSG after gastric banding.

Although PLSG’s efficacy and safety are well-established, RLSG’s outcomes may differ given the anatomical and histopathological changes to the stomach after GB. The rates of surgical complication rates were relatively higher in the RLSG group in the early and late postoperative periods, however the differences were not statistically significant. Other studies have reported insignificant differences in early and late complication rates between PLSG and RLSG [24–28]. Notably, the rates of common major surgical complications after RLSG, such as staple line leaks (2.8%), strictures (1.4%) and bleeding (0.0%), were relatively low in this study. Postoperative bleeding occurs in 2.8% of 1-stage RLSGs and 4.3% of 2-stage RLSGs [29]. Staple line leaks occur in 5.8% of 1-stage RLSG cases and 2.8% of 2-stage RLSG cases [29]. Although RLSG leak rates reported in other studies were comparable to this study’s, no patients in this study required reoperation for leaks.

The low rate of surgical complications after PLSG and RLSG in this cohort mirrors other studies; however, the sample size is underpowered to pick up potential differences in major complications which could potentially occur more frequently in RLSG patients because of their greater operative complexity. RLSGs were conducted in 2 stages with a minimum duration of 2 months between band removal and RLSG. Some studies have suggested utilizing a 2-staged revision approach instead of 1-stage revision to allow inflammation from band removal to abate before RLSG, thereby reducing potential complication risks [27, 28, 30, 31]. Patient reassessment after LAGB removal also allowed the redirection of patients away from RLSG to gastric bypass if there were factors that would increase their likelihood of experiencing poorer functional outcomes.

In terms of functional complications, while more PLSG patients reported early reflux-like symptoms compared to RLSG patients (6.9% vs. 0.0%, *p* = 0.03), these symptoms could be related to differences in oesophageal compliance and sensitivity of PLSG patients with a surgically naïve oesophagus compared to RLSG patients who might be more habituated to oesophageal obstructive symptoms from previous gastric banding. Moreover, this could be due to the exclusion of gastric banding patients who had dysmotility diagnosed at preoperative assessment from undergoing RLSG. In the late postoperative period, while more RLSG patients reported new onset reflux, the difference was insignificant (34.7% vs. 28.5%, *p* = 0.35).

Most comparative studies did not specify the prevalence of GERD after RLSG in the late postoperative period. A French study [27] reported no cases of GERD in both groups postoperatively. In contrast, an Italian study [32] reported significantly higher rates of GERD in the RLSG group (9% mildly symptomatic, 23% severely symptomatic) than the PLSG group (1.3% mildly symptomatic, 3% severely symptomatic) in the long-term. The overall rate of new onset reflux in the late postoperative period (30.6%) is relatively high in this study, but rates of up to 36.0% have been reported in literature [33]. New-onset reflux symptoms after sleeve gastrectomy could be due to several pathophysiological changes, including increased intragastric pressure, esophageal dysmotility, decreased lower esophageal sphincter pressure and delayed gastric emptying [33–37]. While changes to esophageal motility after GB could have predisposed RLSG patients to increased risks of reflux symptoms [38], efforts were made to prevent these patients from undergoing RLSG in this surgical practice.

This study noted poorer WL outcomes with RLSG in the short-term. PLSG patients achieved significantly greater %EWL and %TWL than RLSG patients at up to 36 months, thereafter the difference between the groups diminished. This coincided with the trends of weight regain, evidenced by reductions in ΔBMI, %EWL and %TWL around 18 months. Although such trends suggest comparable WL results between both procedures at middle-term follow-up, these results may not accurately reflect PLSG’s and RLSG’s WL efficacies given the smaller sample sizes at later follow-up, potentially resulting in attrition bias.

Current evidence on the WL outcomes of RLSG and PLSG (Table 5) are conflicting. Two cohort studies have found comparable %EWL between both groups at 2 years [28] and 3 years [39] postoperatively. This contradicts the findings of 2 series [27, 40], which showed consistently significant differences in %EWL in favor of PLSG at all time points up to 6 years follow-up. Other studies that utilized 1-stage RLSG found comparable %EWL between both groups from 1 year [26] up to 5 years [24] postoperatively. A long-term retrospective cohort study [41] found significantly higher percent excess BMI lost in the RLSG group at 6 years and 9 years follow-up. In contrast, a recent cross-matched study [42] found significant higher %EWL in the PLSG group in the short-term but similar %EWL between both groups in the long-term. However, the long-term results may have been influenced by the significant differences in follow-up durations between both groups [42]. Regardless, the long-term differences in WL efficacy between RLSG after GB and PLSG are unknown due to the lack of large, long-term, matched studies.

**Table 5:** Weight loss outcomes of primary and revisional laparoscopic sleeve gastrectomy after gastric banding in 5 retrospective studies

Follow-up period	Studies	%EWL		*p*
		PLSG	RLSG	
3 months	Barrett et al. (2014) [26]	34.7	31.8	0.18
	Alqahtani et al. (2016) [24]	30.5	29.8	0.09
6 months	Barrett et al. (2014) [26]	48.0	41.9	0.07
	Alqahtani et al. (2016) [24]	43.7	44.5	0.26
	Silecchia et al. (2014) [28]	49.8	46.5	0.03
	Goitein et al. (2011) [39]	55.0	37.0	-
9 months	Barrett et al. (2014) [26]	52.0	46.7	0.25
1 year	Barrett et al. (2014) [26]	56.9	47.6	0.09
	Alqahtani et al. (2016) [24]	62.4	60.5	0.10
	Silecchia et al. (2014) [28]	78.2	66.4	<0.01
	Carandina et al. (2017) [40]	67.0	48.5	<0.01
	Goitein et al. (2011) [39]	73.0	53.0	-
2 years	Silecchia et al. (2014) [28]	78.0	78.5	<0.30
	Alqahtani et al. (2016) [24]	73.9	70.4	0.10
	Carandina et al. (2017) [40]	71.6	52.6	<0.01
	Goitein et al. (2011) [39]	84.0	51.0	-
3 years	Alqahtani et al. (2016)	79.1	81.3	0.50
	Carandina et al. (2017) [40]	72.1	51.2	<0.01
	Goitein et al. (2011) [39]	80.0	48.0	-
4 years	Alqahtani et al. (2016) [24]	83.6	79.2	0.37
	Carandina et al. (2017) [40]	69.0	43.4	<0.01
5 years	Alqahtani et al. (2016) [24]	80.4	75.1	0.35
	Carandina et al. (2017) [40]	63.5	37.0	<0.01
6 years	Carandina et al. (2017) [40]	57.2	29.0	0.04

*%EWL* percent excess weight loss; *PLSG* primary laparoscopic sleeve gastrectomy; *RLSG* revision laparoscopic sleeve gastrectomy

The WL outcomes of RLSG after GB in this study (49.0%EWL at 3 years, 39.1%EWL at 5 years) were similar those in a French study (52.6%EWL at 3 years, 37.0%EWL at 5 years) [40]. These results were less favorable than those reported in 2 larger studies (62.6%EWL at 3 years [27]; 75.1%EWL at 5 years [24]). A plausible explanation for this could be differences in patient selection. All RLSG patients in this study underwent revision for IWL and WR, whereas some patients in other studies underwent revision for complications only. Hence, there were differences in patients’ WL history. Weight loss failure with GB in our RLSG group could reflect poorer patient compliance to multidisciplinary intervention or underlying physiology contributing to resistance to WL.

### Limitations

The propensity-score matched study design minimizes the degree of confounding bias compared to cohort studies. However, there could be underlying differences in unobservable or unknown covariates that were not matched for, potentially confounding the results. There were potential selection biases given the study’s retrospective nature and the small sample size of patients from a single institution. The pre-gastric band weights of 22 RLSG patients, who underwent gastric banding at other surgical centers, were unavailable. This could have potentially skewed the baseline characteristics of patients in the RLSG group. Another limitation was the loss to follow-up. Attrition bias could have influenced the differences or similarities in outcomes between the groups. Due to the lack of long-term data, no inferences on the long-term outcomes could be made. Larger long-term studies are needed to gain further insight into the safety and efficacy of RLSG after failed GB. Future studies should also take into consideration patients’ compliance to nutritional management as well as mental health issues which could also influence WL results.

## Conclusion

While RLSG is relatively efficacious in achieving further WL after GB failure in the short-term, the outcomes are poorer compared to PLSG. The poorer weight loss results of RLSG compared to PLSG should be discussed with prospective patients seeking revision bariatric surgery for IWL or WR. Although the risks of surgical and functional complications were not significantly different between RLSG and PLSG patients in this study, adequate preoperative assessments should be conducted to exclude patients with GERD and esophageal dysfunction to reduce the risks of functional complications.

## Appendix

**Table 6:** Baseline characteristics of patients in the primary and revision sleeve gastrectomy groups before and after nearest neighbor propensity score matching (ratio = 2:1, caliper = 0.1, without replacement)

Variables	Unmatched cohort				Matched cohort			
	PLSG (*N* = 991)	RLSG (N = 74)	*p*	*d*	PLSG (*N* = 144)	RLSG (*N* = 72)	*p*	*d*
Propensity Score*	0.07 ± 0.03 (0.0 – 0.18)	0.09 ± 0.03 (0.02 – 0.14)	0.00	-0.56	0.09 ± 0.03 (0.01 – 0.14)	0.09 ± 0.03 (0.02 – 0.14)	0.83	0.00
Age, years*	44.6 ± 12.6 (13.5 – 75.7)	45.7 ± 11.9 (23.1 – 75.4)	0.44	0.10	45.5 ± 11.9 (18.4 – 74.5)	45.3 ± 11.7 (23.1 – 75.4)	0.90	0.02
Preoperative BMI, kg/m^2^*	43.9 ± 7.1 (28.7 – 78.5)	43.0 ± 7.9 (30.1 – 69.2)	0.28	0.12	42.5 ± 6.5 (30.2 – 68.2)	42.7 ± 7.9 (30.1 – 69.2)	0.86	0.03
Sex, *N* (%)								
Female	737 (74.4%)	63 (85.1%)	0.05	0.27	125 (86.8%)	62 (86.1%)	1.00	0.02
Male	254 (25.6%)	11 (14.9%)			19 (13.2%)	10 (13.8%)		
Smoking history, *N* (%)	414 (41.8%)	23 (31.1%)	0.09	0.22	42 (29.2%)	22 (30.6%)	1.00	0.03
GERD, *N* (%)	382 (38.5%)	26 (35.1%)	0.65	0.07	44 (30.6%)	25 (34.7%)	0.64	0.09
Diabetic status, *N* (%)								
No diabetes	648 (65.4%)	56 (75.7%)	0.09	0.23	109 (75.7%)	55 (76.4%)	1.00	0.02
Type 2 diabetes mellitus	150 (15.1%)	13 (17.6%)	0.69	0.07	22 (15.3%)	12 (16.7%)	0.95	0.04
Insulin resistance	189 (19.1%)	5 (6.8%)	0.01	0.37	13 (9.0%)	5 (6.9%)	0.79	0.08
Type 1 diabetes mellitus	4 (0.4%)	0 (0.0%)	1.00	0.09	0 (0%)	0 (0%)	-	<0.01

*Values are expressed as mean ± standard deviation (range)

*N* number of patients; *PLSG* primary laparoscopic sleeve gastrectomy; *RLSG* revision laparoscopic sleeve gastrectomy; *BMI* body mass index; *GERD* gastroesophageal reflux disease
